# The repeatability of superficial retinal vessel density measurements in eyes with long axial length using optical coherence tomography angiography

**DOI:** 10.1186/s12886-018-0992-y

**Published:** 2018-12-17

**Authors:** Mengyang Li, Enzhong Jin, Chongya Dong, Chuan Zhang, Mingwei Zhao, Jinfeng Qu

**Affiliations:** 1 Department of Ophthalmology, Peking University People’s Hospital; Eye diseases and optometry Institute; Beijing Key Laboratory of Diagnosis and Therapy of Retinal and Choroid Diseases, College of Optometry, Peking University Health Science Center, Beijing, 100044 China; 2 Peking University First Hospital, Beijing, 100034 China; 3 Department of Ophthalmology, Peking University People’s Hospital, No. 11 S Ave of XiZhiMen, XiCheng District, Beijing, 100044 People’s Republic of China

**Keywords:** Optical coherence tomography angiography, Myopia, Vessel length density, Perfusion density, Fovea avascular zone, Repeatability

## Abstract

**Background:**

To investigate the repeatability of superficial retinal vessel density measurements in healthy eyes with long axial length (AL) using optical coherence tomography angiography (OCTA).

**Methods:**

There were 60 eyes of 31 volunteers enrolled in this cross-sectional observational study. All subjects underwent OCTA, AL and refraction test. The enrolled eyes were divided into the long AL group (26 mm ≤ AL < 28 mm) and normal AL group (22 mm ≤ AL < 26 mm). The vessel length density (VLD), perfusion density (PD), and fovea avascular zone (FAZ) of the superficial retinal vessel were evaluated. Repeatability was assessed by intraclass correlation coefficients (ICCs) and Bland-Altman analysis. Pearson’s r correlation was used to analyze the relation of AL and the absolute difference between two measurements.

**Results:**

The 3 × 3 mm scan pattern showed good repeatability with all ICCs over 0.7. For all parameters of all scan patterns, the ICCs of the normal AL group were distinctly higher than those of the long AL group; this finding was also confirmed by Bland-Altman analysis. The correlation analysis of AL and repeatability of OCTA parameters showed significant negative correlations between the ALs and repeatability of VLD in 6 × 6 mm inner ring (*r*^2^ = 0.13, *p* = 0.01), VLD in 6 × 6 mm outer ring (*r*^2^ = 0.09, *p* = 0.02) and PD in 6 × 6 mm outer ring (*r*^2^ = 0.08, *p* = 0.03).

**Conclusions:**

The AL and the scanned area will both affect the repeatability of superficial retinal vessel density measurements in OCTA.

**Electronic supplementary material:**

The online version of this article (10.1186/s12886-018-0992-y) contains supplementary material, which is available to authorized users.

## Introduction

Optical coherence tomography angiography (OCTA) is an advanced imaging technique that allows depth resolved study of retinal microvascular networks. Compared with traditional angiography using contrast agents, OCTA can obtain more subtle macular structure details [1]. Since high myopia has become a prominent worldwide problem especially in Asia [2], understanding the alteration of retinal and choroidal vascular network in the myopia population using OCTA was of great significance. Previous studies have performed many quantitative analyses of retinal vascular network using OCTA in myopic eyes [3–5].

The superficial capillary plexus with surrounding pericytes is a component of the inner blood retina barrier, and changes in this structure play an important role in formation of macular edema in diabetic retinopathy, retinal vein occlusion, macular degeneration and other vascular diseases [6, 7]. ZEISS AngioPlex Metrix software provides automatic quantitative analysis of retinal vessels in the superficial layer, including the fovea avascular zone (FAZ), vessel length density (VLD) and perfusion density (PD), but the repeatability remains unclear. The FAZ, VLD and PD of the superficial retinal vasculature have been reported to have high repeatability but can be affected by many factors, such as scan order [8]. In our clinical practice, we have noticed the difficulty in acquiring high-quality images of some patients with high myopia. Therefore, we intend to explore the correlation between the repeatability of OCTA parameters and AL of healthy young adults for better understanding and interpretation of vascular perfusion in patients with axial myopia.

In this study, we compare the repeatability of the FAZ, VLD and PD of the superficial retinal vasculature between eyes with long AL and normal AL. Parameters from 3 × 3 mm and 6 × 6 mm scans were analyzed separately.

## Materials and methods

### Participants

This cross-sectional study was approved by the Peking University People’s Hospital Review Board and adhered to the tenets of the Declaration of Helsinki and Health Insurance Portability and Accountability Act. Written informed consent was obtained from all individual participants included in the study.

Sixty eyes of 31 healthy subjects were enrolled in this study from May 1, 2017 to October 10, 2018. The inclusion criteria were as follows: (1) age between 20 and 35 years old; (2) no history of systemic diseases; (3) no known eye disease except for refractory error; (4) no ocular media opacity, which was confirmed by slit-lamp examination (SLE); (5) best-corrected visual acuity (BCVA) no worse than 20/20; (6) no ocular surgery history, including refractory surgery. The exclusion criteria were as follows: (1) unable to acquire good quality images after repeated measurements or poor fixation; (2) refractive error greater than − 10.00 spherical diopters or − 2.00 cylindrical diopters; (3) AL longer than 28 mm. The enrolled eyes were divided into the long AL group (AL ≥ 26 mm) and normal AL group (AL < 26 mm).

All subjects underwent BCVA, SLE, refraction test, AL measurement and OCTA. AL measurement was performed using IOLMaster (Zeiss 500; Carl Zeiss Meditec, Inc., Dublin, CA).

### Optical coherence tomography angiography image acquisition

OCTA images were acquired using Cirrus HD-OCT model 5000 (AngioPlex software, version 10.0; Carl Zeiss Meditec, Inc) in a dark room. All measurements were performed by one operator (M.L.) under FastTrac mode. For each eye, a 3 × 3 mm scan and a 6 × 6 mm scan centered on the fovea were acquired. Each scan was repeated at least twice for repeatability analysis. Automated OCT segmentation was performed on qualified images and manual adjustment was applied when segmentation error occurs. The qualifying images were defined satisfying the following requirements: (1) signal strength (SS) ≥7, (2) no more than one blink or motion artifact, and (3) the macular fovea remained in the center of the scanned area. The difference of SS between each two repeated scans should not lager than 1. Based on these default settings, the superficial capillary plexus (SCP) en face image was segmented with an inner boundary at internal limiting membrane (ILM) and an outer boundary at the junction of inner plexiform layer (IPL) and inner nuclear layer (INL) [9].

### Vascular parameters measurement

**Fig. 1:**
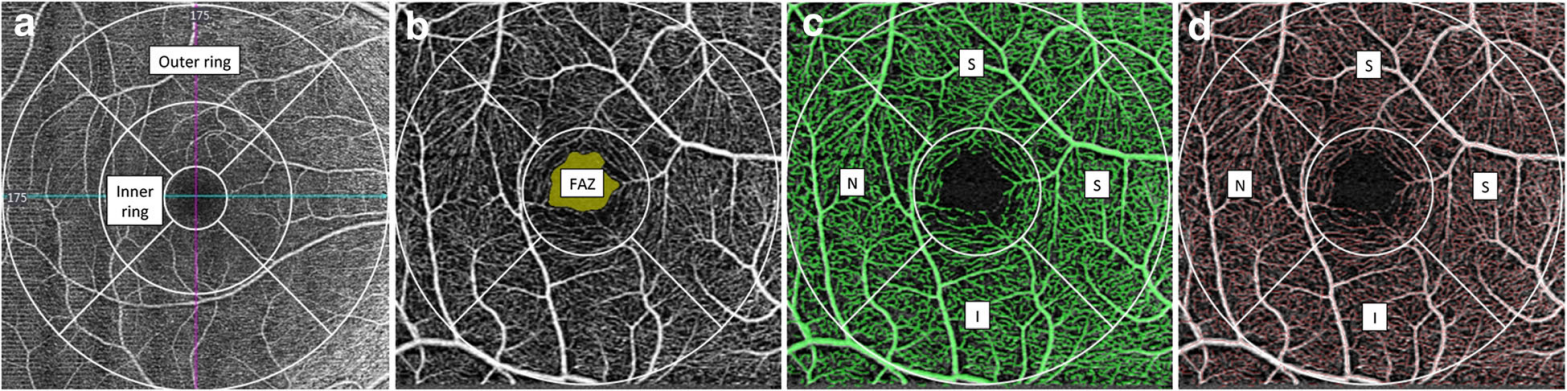
Superficial vessel plexus en face images of OCTA. **a** The inner ring and outer ring of the 6 × 6 mm scan pattern. **b** The FAZ area automatically recognized by the software on the 3 × 3 mm scan pattern; **c** The perfused vasculature (marked as green) automatically segmented to calculate PD; **d** The skeletonized vasculature (marked as red) automatically segmented to calculate VLD

The FAZ area, VLD and PD of SCP within ETDRS 3 × 3 mm inner ring, 3 × 3 whole grid, 6 × 6 mm inner ring, 6 × 6 mm outer ring and 6 × 6 whole grid was evaluated separately (Fig. 1). FAZ area were segmented automatically and manually adjust if necessary, and then measured respectively in both 3 × 3 and 6 × 6 mm scans by AngioPlex software automatically (Fig. 1b). PD was defined as the total area of perfused vasculature per unit area in a region of evaluation (Fig. 1c). VLD was defined as the total length of skeletonized perfused vasculature per unit area in a region of evaluation (Fig. 1d).

### Statistical analysis

Intraclass correlation coefficients (ICCs) with 95% CIs were used to evaluate the repeatability of PD, VLD and FAZ between two consecutive scans of the same pattern and also between the two groups. Considering we include both eyes of subjects in our study, the ICCs were further adjusted for inter-eye correlation using a mixed-design analysis of variance model. Agreement between two measurements was also evaluated by Bland-Altman plots. Pearson’s r correlation test was used to analyze the relation of ALs and absolute differences between two measurements. The absolute difference was defined as |m_1_-m_2_| in which m1 was the first measurement result of PD, VLD or FAZ, and m_2_ was the second measurement. SPSS (version 23.0, IBM Corporation, Armonk, NY) was used for ICCs calculation. Bland-Altman analysis and Pearson’s r correlation test were performed using GraphPad Prism (version 6.00 for Mac, GraphPad Software, La Jolla, California, USA). *p* < 0.05 was considered significant.

## Results

A total of 31 subjects and 62 eyes were enrolled in this study. Two eyes were excluded due to poor SS, the remaining 60 eyes were included in the analysis. The demographics of the enrolled subjects are shown in Table 1. The raw data of VLD, PD and FAZ values for 3 × 3 mm and 6 × 6 mm were shown in Additional file 1. Mann-Whitney test was used to compare the demographics of the normal AL group (*n* = 32) with those of the long AL group (*n* = 28). The mean age of all subjects was 25.61 ± 3.00 years old (range 22–35 years), and no significant difference was shown between the two groups (*p >* 0.05). The ALs and spherical diopter were significantly different between the two groups (*p* < 0.05).

**Table 1:** Demographic and clinical data of the enrolled subjects

Variables	Value			
	22 mm ≤ AL < 26 mm	26 mm ≤ AL < 28 mm	Total	*p*-value
Number	32	28	60	–
Mean age (±SD)	25.87 (±2.73)	25.32 (±3.30)	25.61(±3.00)	0.20
Mean AL (±SD)	24.73(±0.68)	26.73 (±0.52)	25.66 (±1.17)	< 0.01
Mean diopter (±SD)	−3.27(±1.57)	−6.36(±1.70)	−4.71(±2.24)	< 0.01

*Abbreviations*: *SD* standard deviation, *AL* axial length

**Fig. 2:**
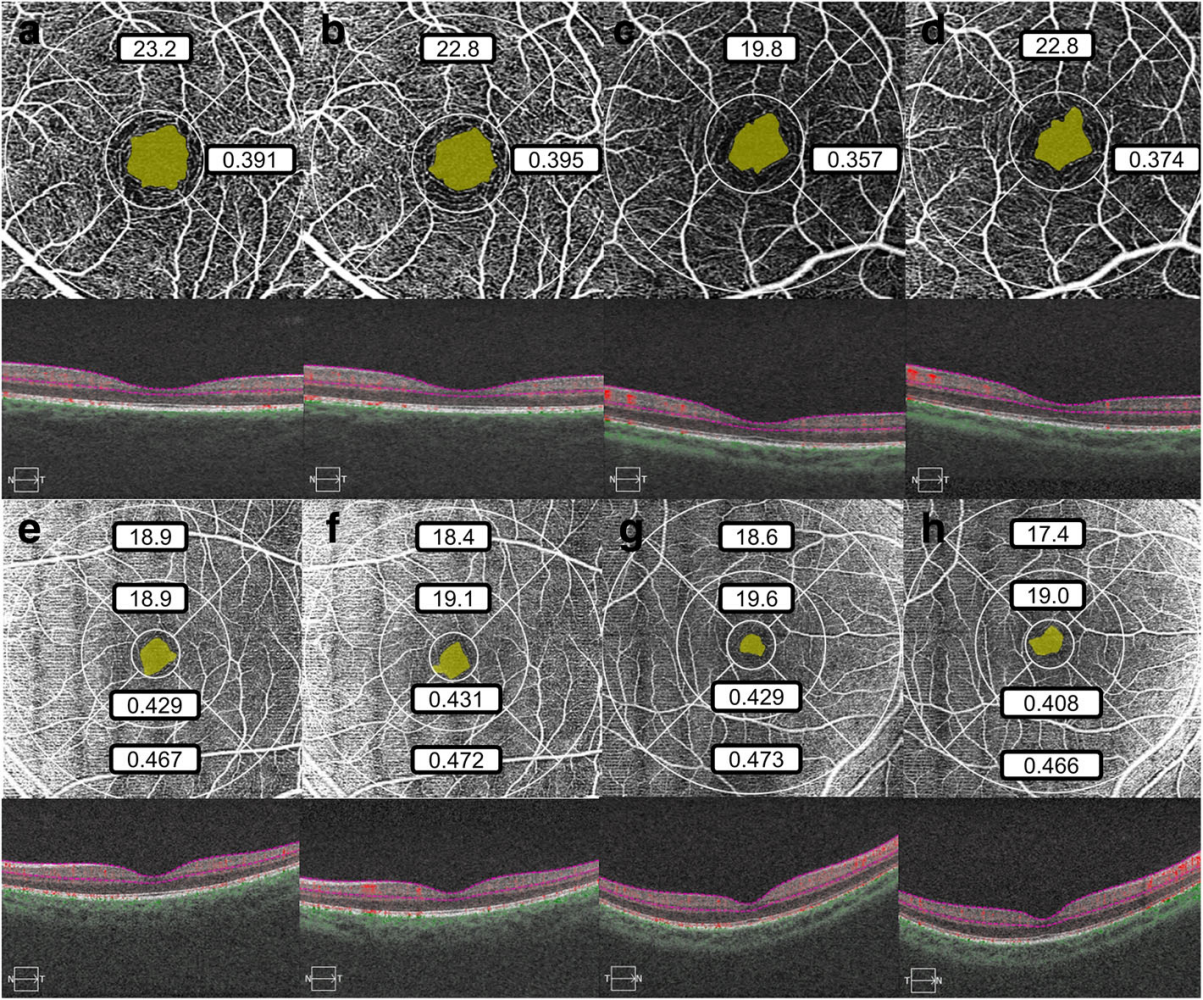
The representative images of OCTA images of normal and long axial length eyes showed the comparation of VLD, PD and FAZ area between the two groups. **a** and **b** showed two repeated OCTA 3 × 3 mm SCP en face image and b-scans of a 25-year-old male with an axial length of 24.51 mm. **c** and **d** showed two repeat 3 × 3 mm SCP en face images of a 35-year-old male with an axial length of 27.34 mm. The yellow area was the automatically segmented foveal avascular zone. The upper and right numbers were VLD and PD in the inner ring respectively. **e** and **f** showed two repeated OCTA 6 × 6 mm scans of a 26-year-old female with an axial length of 23.58 mm; **g** and **h** showed 6 × 6 mm scans of a 35-year-old male with an axial length of 27.27 mm. The yellow area was the automatically segmented foveal avascular zone. The top, upper middle, lower middle and bottom numbers represent VLD in the outer ring, VLD in the inner ring, PD in the inner ring and PD in the outer ring. Noticed the differences in all metrics between two repeated scans and lower repeatability in long axial eyes

The representative OCTA images of both normal and long AL group were demonstrated in Fig. 2, which showed the comparation of VLD, PD and FAZ area outline between the two groups. The ICCs and 95% confidence intervals of VLD, PD and FAZ are shown in Table 2. There were very subtle changes of ICCs after adjusted for inter-eye correlation using a mixed-design analysis of variance model (ICC_*adj*_ in Table 2). Therefore, we believed that the involvement of both eyes will not affect the repeatability test in this study. For all parameters of all scan patterns, the ICC of the normal AL group was much higher than that of the long AL group, with the biggest difference of VLD in 6 × 6 inner ring and smallest difference of PD in 3 × 3 inner ring and FAZ area in 6 × 6 scan. In normal AL group, the repeatability was very good (ICC > 0.9) in VLD and PD in 3 × 3 inner ring and FAZ area in 3 × 3 mm scan; was good (ICC > 0.7) in VLD and PD in 6 × 6 inner ring; was moderate (ICC > 0.4) in VLD and PD in 6 × 6 outer ring and FAZ area in 6 × 6 mm scan.. In long AL group, no parameters has ICC larger than 0.9, the repeatability was good in VLD and PD in 3 × 3 inner ring and FAZ area in 3 × 3 mm scan; was moderate in VLD and PD in 6 × 6 inner and outer ring and FAZ area in 6 × 6 mm scan. For VLD and PD in inner ring and FAZ area, the repeatability was higher in 3 × 3 mm scan than in 6 × 6 mm scan.

**Table 2:** Statistical analysis results: ICC of VLD, PD and FAZ

Scan patterns and metrics		22 mm ≤ AL < 26 mm		26 mm ≤ AL < 28 mm		Total	
		ICC	ICC_*adj*_	ICC	ICC_*adj*_	ICC	ICC_*adj*_
VLD	3 × 3 inner	0.90(0.81–0.95)	0.91	0.78(0.56–0.88)	0.75	0.83(0.74–0.90)	0.83
	6 × 6 inner	0.76(0.56–0.87)	0.76	0.41(0.05–0.67)	0.41	0.54(0.31–0.70)	0.54
	6 × 6 outer	0.61(0.34–0.79)	0.61	0.43(0.08–0.69)	0.43	0.50(0.28–0.66)	0.50
PD	3 × 3 inner	0.90(0.81–0.95)	0.90	0.80(0.63–0.90)	0.81	0.86(0.77–0.91)	0.85
	6 × 6 inner	0.71(0.49–0.85)	0.71	0.43(0.07–0.68)	0.43	0.52(0.31–0.68)	0.52
	6 × 6 outer	0.55(0.26–0.75)	0.54	0.40(0.04–0.67)	0.40	0.46(0.23–0.64)	0.46
FAZ	3 × 3	0.96(0.92–0.98)	0.96	0.86(0.73–0.94)	0.80	0.92(0.87–0.95)	0.92
	6 × 6	0.69(0.46–0.84)	0.69	0.62(0.34–0.80)	0.60	0.65(0.48–0.77)	0.67

ICCs of VLD, PD and FAZ in two groups measured by 3 × 3 mm and 6 × 6 mm scan patterns are presented in Table 2. The columns of ICC_*adj*_ showed the ICCs adjusted for inter-eye correlation using a mixed-design analysis of variance model

*Abbreviations*: *VLD* vessel length density, *PD* perfusion density, *FAZ* fovea avascular zone, *AL* axial length, ICC_*adj*_ adjusted ICC

**Fig. 3:**
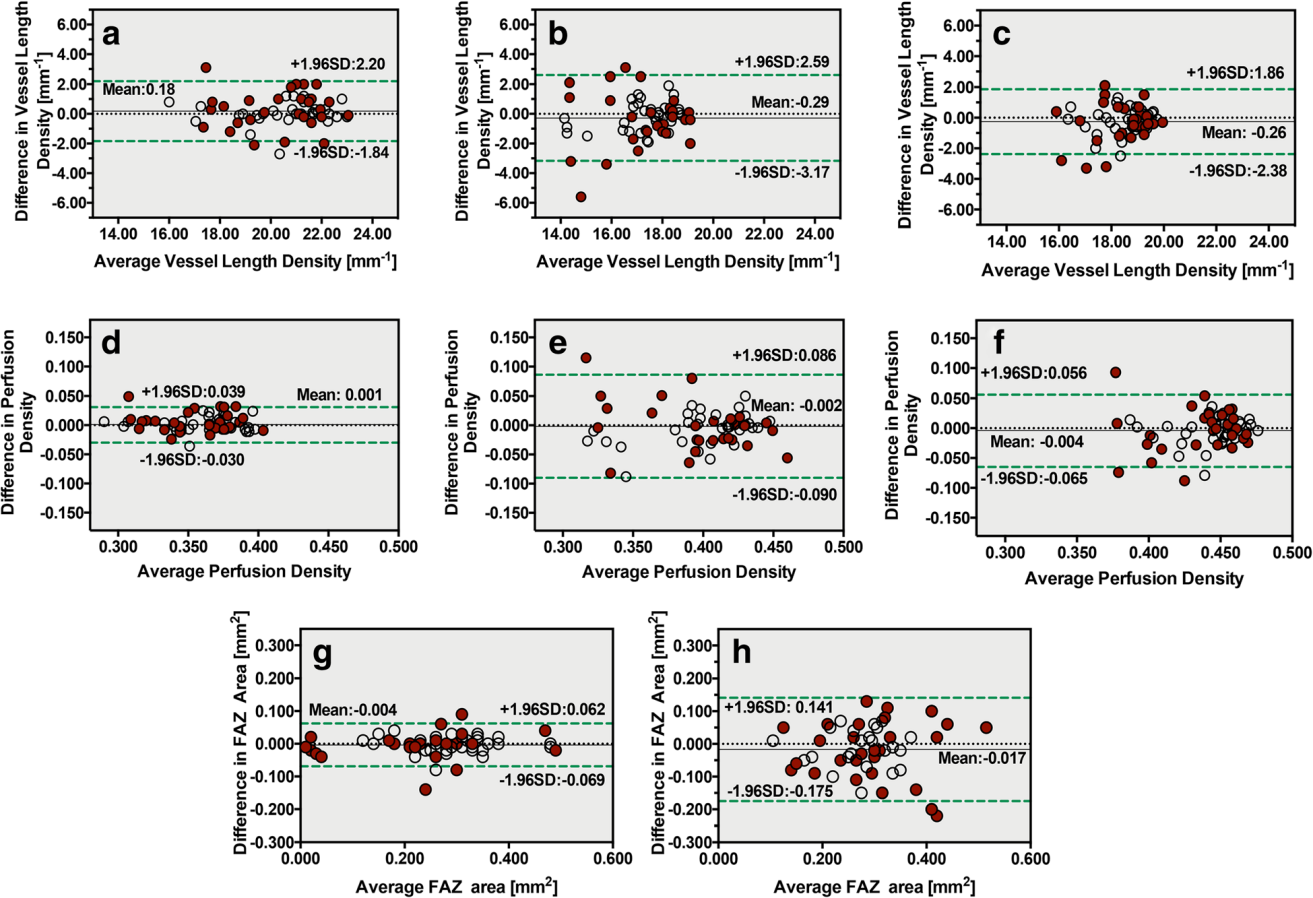
Bland-Altman analysis of VLD, PD and FAZ in two different scan patterns. All of the values from the long AL group are marked as red dots, and values from the normal AL group are marked as empty circles. **a** Bland-Altman analysis of VLD in 3 × 3 mm inner ring. **b** VLD in 6 × 6 mm inner ring. **c** VLD in 6 × 6 mm outer ring. **d**, **e**, **f** showed Bland-Altman analysis of PD. **d** Bland-Altman analysis of PD in 3 × 3 mm inner ring. **e** PD in 6 × 6 mm inner ring. **f** PD in 6 × 6 mm outer ring. **g** Bland-Altman analysis of FAZ in 3 × 3 mm scan. **h** Bland-Altman analysis of FAZ in 6 × 6 mm scan

Bland-Altman analysis of VLD, PD and FAZ is shown in Fig. 3. The mean differences in VLD, PD and FAZ area are depicted by black horizontal lines in the graph. Two green dashed horizontal lines indicate the mean difference ± 1.96 SD, which are also referred to as limit agreements (LAs), representing the range of values for the differences between the two measurements that could be expected 95% of the time. All of the values from the long AL group are marked as red dots.

For VLD, the range of LA was smallest in the 3 × 3 mm inner ring (lower and upper bounds of − 1.84 and 2.20 mm^− 1^) and biggest in the 6 × 6 inner ring (lower and upper bounds of − 3.17 and 2.59 mm^− 1^). The number of eyes beyond LAs of VLD in the 3 × 3 mm inner ring was 4 eyes in long AL group and 1 eye in normal AL group. The number of eyes beyond LAs of VLD in the 6 × 6 mm inner ring was 6 eyes in long AL group and no eye in normal AL group. The number of eyes beyond LAs of VLD in the 6 × 6 mm outer ring was 4 eyes in long AL group and 1 eye in normal AL group.

For PD, the range of LA was smallest in the 3 × 3 mm inner ring (lower and upper bounds of − 0.030 and 0.039 mm^− 1^) and biggest in the 6 × 6 inner ring (lower and upper bounds of − 0.090 and 0.086 mm^− 1^). The number of eyes beyond LAs of PD in the 3 × 3 mm inner ring was 1 eye in each group. The number of eyes beyond LAs of PD in the 6 × 6 mm inner ring was 1 eye in long AL group and no eye in normal AL group. The number of eyes beyond LAs of PD in the 6 × 6 mm outer ring was 3 eyes in long AL group and 1 eye in normal AL group.

For FAZ, the range of LA was smaller in the 3 × 3 mm scan (lower and upper bounds of − 0.069 and 0.062 mm^− 1^) and biggest in the 6 × 6 mm scan (lower and upper bounds of − 0.175 and 0.141 mm^− 1^). The number of eyes beyond LAs of PD in the 3 × 3 mm scan was 3 eyes in long AL group and 1 eye in normal AL group. The number of eyes beyond LAs of PD in the 6 × 6 mm scan was 2 eyes in long AL group and no eye in normal AL group.

**Fig. 4:**
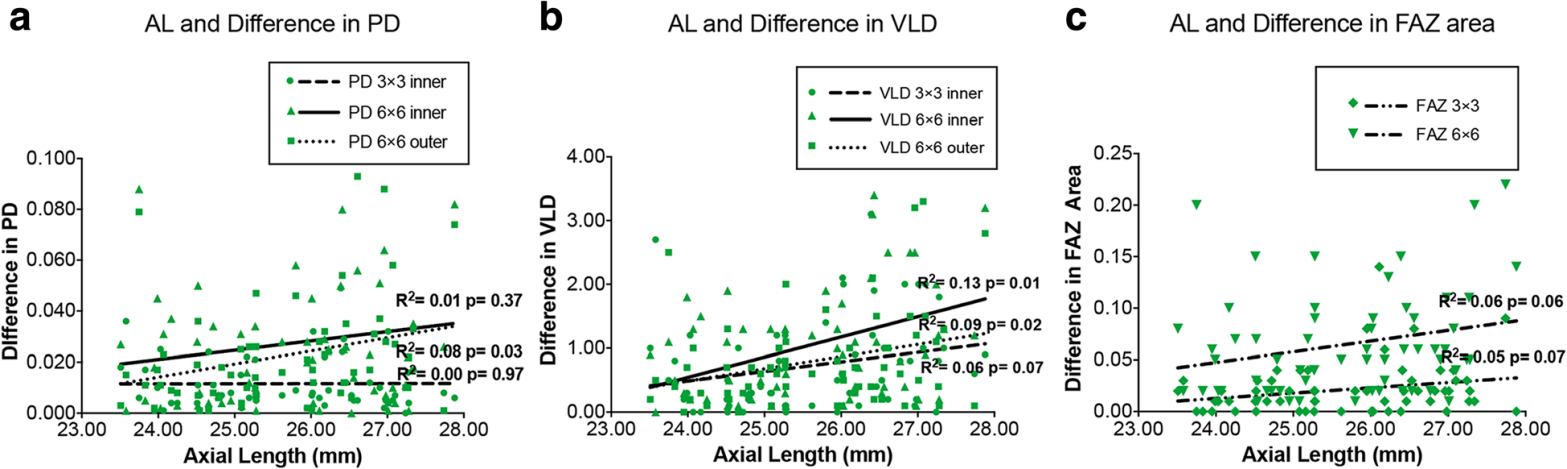
Relationship between AL and the absolute difference between two constant measurements in PD, VLD and FAZ area. **a** AL and absolute difference in PD. **b** AL and absolute difference in VLD. **c** AL and absolute difference in FAZ area

The correlation between AL and the absolute difference of two measurements is shown in Fig. 4. The scatter plots and fitting lines indicated significant negative correlations between the ALs and repeatability of VLD in 6 × 6 mm inner ring (*r*^2^ = 0.13, *p* = 0.01), VLD in 6 × 6 mm outer ring (*r*^2^ = 0.09, *p* = 0.02) and PD in 6 × 6 mm outer ring (*r*^2^ = 0.08, *p* = 0.03). Comparing to VLD, the repeatability of PD was less affected by AL in both 3 × 3 mm and 6 × 6 mm scans. There was also borderline statistically significant negative correlation between VLD in 6 × 6 mm outer ring (*r*^2^ = 0.06, *p* = 0.07), FAZ area in 6 × 6 scan (*r*^2^ = 0.06, *p* = 0.06) and 3 × 3 scan (*r*^2^ = 0.05, *p* = 0.07).

## Discussion

Myopia is one of the most prevalent eye disorders and is estimated to affect 1.5 billion people worldwide. Previously, with the development of OCTA, many researchers have focused on quantification of macular vascular density using OCTA. Changes in the superficial and deep retinal microvasculature as well as choroidal capillaries have been found in high myopia patients [4, 10–12]. These changes could be explained by longer axial length and thinner choroid but the repeatability of these measurements should be considered before we draw any conclusion. Unfortunately, most of previous studies did not report their repeatability of quantification parameters. The variation of the use of build-in automatic algorism or custom algorism and the application of magnification correction in different studies makes it difficult to interpret the result from different studies.

To the best of our knowledge, this study is the first to explore the repeatability of the FAZ, VLD and PD of SCP between eyes with long AL and normal. Many previous studies revealed the good repeatability of Optovue Avanti OCTA [13–16] and other devices [17–19] in normal AL eyes. For the ZEISS device, researchers also reported good repeatability of FAZ in eyes with spherical refraction within − 3.00D [20]. However, their work was based on manual segmentation and algorism using ImageJ, which inevitably introduces human error into the evaluation. Another study using built-in AngioPlex software for automatic quantitative analysis report excellent repeatability in normal eyes, with a greater ICC for the 3 × 3 mm scan pattern [8]. The ICCs of vessel density and FAZ area in those studies were remarkably higher than the repeatability of them in all subjects in our results, probably because the average AL in our study (25.66 mm) was higher than theirs.

For all parameters of all scan patterns, the ICCs of the normal AL group were distinctly higher than those of the long AL group; this finding was also confirmed by Bland-Altman analysis. The correlation analysis of AL and repeatability of OCTA parameters showed significant or borderline significant negative correlations between the ALs and repeatability of almost all parameters except PD in 3 × 3 mm inner ring. This may be explained by the attenuation of vascular signal in long AL eyes or real reduction of superficial macular microvascular complex in long AL eyes. With the progression of myopia, the elongation of the eyeball stretches the retinal tissue, some very narrow microvascular may become undetectable now and then in the repeat scans.

In general, quantitative analysis of vessel density of the inner ring of the 3 × 3 mm scan pattern had the best repeatability, with an ICC_*adj*_ of 0.90 and 0.91 for VLD and PD, respectively. The ICC of FAZ area in 3 × 3 mm scan was the highest (0.96) among all parameters. These indicate that the scan area also has influence on the repeatability of the parameters we studied. From the Bland-Altman analysis of VLD, PD and FAZ, we can notice that the distribution of VLD in the 6 × 6 mm inner ring moved to the smaller side of X axial and become more disperse comparing to 3 × 3 mm inner ring. A possible explanation is the decreased resolution of microvascular network in large scanned area. Thus, cautions should be raised to interpret quantitative measurement from different scan area. For the evaluation of FAZ, we recommend the 3 × 3 mm scan for better repeatability.

Our study has the following limitations: relative small sample size; all of the images were obtained from one device to avoid variability but the result was also limited to certain device; we limited our subjects to healthy young adults with an age range of 22–35 years to decrease the effect of age and potential age-related vascular diseases, particularly in FAZ area [21, 22]. Thus, further studies are needed to reveal the effect of AL in older population.

## Conclusions

In conclusion, The AL and the scanned area will both affect the repeatability of superficial retinal vessel density measurements in OCTA. The elongation of AL lowered the repeatability of superficial retinal vessel density and FAZ measurements in OCTA. For the same parameter, 3 × 3 mm scan pattern has better repeatability than that of 6 × 6 mm scan pattern. The 6 × 6 mm scan pattern was more affected by AL. Cautions should be raised when comparing microvascular parameters from different scan pattern or from patients with different AL.

## Additional file

Additional file 1: **Table S1.** Raw data of OCTA measurements. This file includes VLD, PD and FAZ values for 3 × 3 mm and 6 × 6 mm scans of all the subjects in this study. (XLSX 32 kb)

## Abbreviations

AL: axial length
ETDR: Early treatment of diabetic retinopathy
FAZ: Fovea avascular zone
ICC: Intraclass correlation coefficient
ICC_*adj*_: Adjusted ICC
OCTA: Optical coherence tomography angiography
PD: Perfusion density
SCP: Superficial capillary plexus
SD: Standard deviation
SS: Signal strength
VLD: Vessel length density
